# Quantitative Evaluation of TOD Performance Based on Multi-Source Data: A Case Study of Shanghai

**DOI:** 10.3389/fpubh.2022.820694

**Published:** 2022-02-21

**Authors:** Dan Qiang, Lingzhu Zhang, Xiaotong Huang

**Affiliations:** ^1^Department of Architecture, College of Architecture and Urban Planning, Tongji University, Shanghai, China; ^2^Key Laboratory of Ecology and Energy-Saving Study of Dense Habitat, Tongji University, Shanghai, China

**Keywords:** transit-oriented development (TOD), TOD performance, pedestrian-oriented, multi-source urban data, station-city integration

## Abstract

Transit-oriented development (TOD) has been widely adopted as a primary urban planning strategy to better integrate transit and land use; further, the pedestrian-oriented perspective has been receiving increasing attention. However, most studies so far have only focused on few features and fail to capture comprehensive perceptions in the transportation (T), pedestrian-oriented accessibility (O), and urban development (D) dimensions. New emerging urban datasets provide a more refined and systematic approach to quantify the characteristics of metro station areas. This study offers a more efficient and convenient process and comprehensive approach to measure TOD performance. With a combination of traditional data collected by an official department, high-resolution open data, and innovative technology, large-scale analyses of 347 metro stations in Shanghai were conducted. Fifteen indicators for T, O, and D were chosen to categorize TOD performance into five clusters. Radar charts, boxplots, and colored maps were used to display numerous quantitative factors for each type. Combining the results with the Shanghai Comprehensive Plan (2017–2035) showed that the majority of Cluster 4 is located at the center of the Five New Towns. The correlation analysis between ridership and TOD performance showed that the transportation dimension indicator has a strong correlation with daily ridership, followed by the O and D indicators. Moreover, ridership per capita was found to be affected by resident density, employment density, O value, and D value, whereas no significant correlation was found between ridership per capita and T value. Population plays a pivotal role in metro passenger traffic, indicating ridership per capita had a high, strong correlation with resident density, with *R* = 0.658 for weekdays and *R* = 0.654 for weekends. This study reinterpreted the node-place method and 5Ds framework, resulting in a renewal method with new datasets and analysis tools. It contributes to providing pedestrian-oriented TOD planning methodology for urban planners and policymakers by combining T, O, and D dimensions and visualizing the results with current urban planning.

## 1. Introduction

China has undergone urbanization at an unprecedented rate over the last few decades. According to the National Bureau of Statistics of China, more than 60% of Chinese residents lived in cities in 2020, and this percentage is expected to rise to 70% by 2035 (1, 2). The cities' general expansion usually leads to large-scale construction of transportation infrastructure as a result of the increasing travel distance (3, 4). For example, urban rail transit is an effective alternative mode that can help mitigate the negative effects of rapid urbanization on the urban transportation system (5–7). Meanwhile, using transit and land-use integration (or “station-city integration”) to develop urban rail transit is one of the most effective strategies, and is frequently endorsed by policymakers (8–10). Transit-oriented development (TOD) is a common response to this integration (11, 12), as it encourages maximizing urban development around public transportation stations and forming high-density communities with diverse functions and walkable environments (13, 14).

In recent years, China has widely adopted TOD as a primary urban planning strategy (15, 16). However, there is still significant progress to be made to achieve efficient TOD, due to the slow growth of theory and practice. In most of China's recent planning strategies, transit station characteristics have received overwhelming attention. In addition, profit-driven land development and fragmentary management have created institutional obstacles to integrating land use and transit (17). Therefore, governments and developers should sufficiently promote the concept of TOD in local contexts and improve their comprehensive understanding of this concept to better integrate transit and land-use (18–20).

Most recently, a rapid increase in quantitative analytical tools and new urban data have provided new research potential in urban transport research. In China, many pioneering studies utilized big data to access daily travel and activities at fine-grained spatial networks (17, 21), and to understand how they interact with social, environmental, and urban forms (22, 23). In terms of evaluating urban structure and function, some researchers identified work and home locations and commuting patterns (24–27), while others revealed the urban polycentric structure and function distribution (28–31). Nevertheless, the application of big data for measuring TOD performance has seldom been studied in China (32).

TOD performance can measure the extent of the existing integration of transit and land use, as well as other attributes, such as pedestrian friendliness, urban form, and quality of public facilities around TOD sites (33, 34). Although several studies have generated many insights on quantifying TOD performance, little research has explored how new urban data could furnish unexpected outcomes and improvement. Multi-source urban data, including points of interest (PoIs), location-based service data, street view images (SVIs), and built environment data, offer a larger and more representative sample and a finer scale of spatiotemporal resolution (35, 36). New technologies, such as machine learning algorithms and Spatial Design Network Analysis (sDNA) based on ArcGIS, provide novel analysis methods for quantifying the characteristics of road and pedestrian networks (37, 38). This can help overcome some limitations of traditional data, including labor consumption, small samples, and low frequency.

In this context, Zhou analyzed a case of 167 metro stations in Shenzhen, combining traditional and non-traditional data (e.g., social media data, digital maps, PoIs, interviews, and surveys) to propose new indicators of TOD performance and its relationship to spatial and behavior characteristics (39). Nonetheless, this previous study mostly focused on transportation behavior on a citywide scale, paying less attention to pedestrian-oriented urban design as well as transit and land use integration. In addition, other quantitative studies have rarely examined TOD types and variations in China to further optimize future urban planning.

Thus, exploring how metro stations can be integrated into urban spaces under the concept of station-city integration, studying the relationship between transit nodes and urban places in-depth, and developing a quantitative synergy evaluation index *via* multi-source urban data are all crucial components of prioritizing sustainable transportation and realizing digital management.

The remainder of the article is organized as follows. Section 2 provides a review of the literature review on TOD performance, node-place model, and human-oriented indicators. Section 3 introduces the methods—the city sample, data, and analysis. Section 4 presents the results on five TOD types. Section 5 provides a discussion on urban planning implications and contributions. Finally, Section 6 presents conclusions and limitations.

## 2. Literature Review

Previous researchers have found that TOD typologies contribute to targeted strategies for TOD promotion (40, 41), the identification of prevalent issues (42, 43), and investment estimates (44). During the late 20th century, many researchers suggested the idea of mixed-use, compact developments with pedestrian-friendly built environments. The “3Ds” (45)—density, diversity, and design—are commonly regarded as the most fundamental indicators for evaluating the TOD performance of railway station areas. However, by combining destination accessibility and distance to transit (46), the “5Ds” became more systematic standards.

The node-place theory provides a practical method for evaluating TOD performance based on travel demand (“node”) and land use (“place”) (47), which subsequent studies have referred to as the “T” (node) and “D” (place) dimensions, respectively (48). A node index was created using the connection, frequency, and diversity of transport services, while a location index was developed using factors such as the functional mixture, number of inhabitants, or number of employees. Five TOD typologies (i.e., balance, stress, dependency, unsustained place, and unsustained node) were identified when two indices were converted into an XY diagram. The station is defined as an “unsustained node” when T exceeds D. It is an “unsustained place” when D surpasses T. The node-place theory is capable of and effective in assessing the balance between transportation supply (T) and surrounding urban development (D) under the station-city integration concept.

However, locations in the node-place method were criticized for being Transit Adjacent Development (TAD), rather than TOD (49, 50). Accordingly, a significant amount of research has introduced numerous modifications and extensions. Table 1 summarizes selected studies on the quantification of TOD performance. Notably, of the 27 publications, 14 referred to walking and pedestrian indicators. Among them, several researchers had introduced a new dimension to extend the classic “node-place” model. Vale (50) proposed the pedestrian shed ratio as a measure of pedestrian friendliness. Based on this, Lyu (48) established an additional “Oriented” (O) dimension of functional and morphological characteristics and selected 18 out of 94 systematic variables to assess Chinese context-based TOD in Beijing. Recent studies have extended “node-place” model to “node-tie-place” model (51) and “node-functionality-place” model (52) by adding functionality indicators (51, 52). Moreover, several previous studies have also emphasized pedestrian-oriented perspective as an important dimension in transit station areas (11, 39, 53–58). Nevertheless, pedestrian-friendly dimension quantitative studies are rarely conducted to comprehensively examine the TOD performance.

**Table 1:** Comparison of the indicators used in the reviewed literature to measure TOD performance.

	**Literature**	**TOD indicators**					**Data sources**
		**T (transportation)**		**O (pedestrians)**	**D (development)**		
01	Bertolini (47)	- Number of directions served by train - Daily frequency of train services - Number of train stations within 45 min of travel - Number of directions served by bus, tram, and underground - Daily frequency of bus, tram, and underground - Distance from the closest motorway access by car - Car parking capacity - Number of free-standing bicycle paths - Bicycle parking capacity			- Population around the station - The number of workers in Cluster 1: services and administration - The number of workers in Cluster 2: retail, hotel, and catering - The number of workers in Cluster 3: industry and distribution - The number of workers in Cluster 4: education, health, and culture - Degree of multifunctionality		Not mentioned
02	Reusser et al. (41)	- Directions served by train - Frequency of train services - Number of stations within 20 min of travel - Number of directions other public transportation (bus and tram) - Daily frequency other public transport - Distance to town center	- Distance from the closest motorway access - Car parking capacity - Bicycle access - Bicycle parking capacity - Passenger frequencies - Type of train services - Staffing		- Population - Number of workers per economic sector [4 clusters, the same as Bertolini (47)] - Degree of functional mix - Commercial services degree - Conference rooms and educational facilities degree		- Swiss Federal Railway's station database - 2,000 Swiss census of the population - 2,001 Swiss census of enterprises - Digital maps
03	Gonçalves and Portugal (66)	The same as Bertolini (47), except that the research scope was different for some indicators			- Number of residents - Number of workers in the secondary sector - Number of workers in the tertiary sector - Degree of functional mix		Not mentioned
04	Cervero and Murakami (67)				- Building area (in gross floor area) by using - Scale (total gross floor area) - Density and verticality - Mix-use attributes		Not mentioned
05	Srikanth (68)	- Access to BRT - Street network		- Pedestrian network - Green/open spaces	- Density of jobs - Density of the population - Ratio of jobs to population	- Commercial index - Dissimilarity index - Social distribution	- GIS data - Interview data
06	Chorus and Bertolini (69)	- Number of train connections - Number of bus connections - Type of train connections - Proximity to CBD by rail - Number of bus lines departing from the station			The same as Bertolini (47)		- Spatial data-sets (Japanese GIS website)
07	Zemp et al. (70)	- Passenger frequencies on weekends compared to weekdays - Number of reachable railway stations in 20 min - Number of departing intercity trains - Number of departing regional trains - Number of departing buses - Main station of a regional center			- Number of jobs - Number of residents - Average distance to jobs and residents - Arriving tourists per 1,000 residents of the municipality		- Transportation timetable data - Face-to-face interviews - Swiss Federal Bureau of Statistics data - Data from Reusser's research
08	Atkinson Palombo and Kuby (71)	- Whether or not a station has a park-and-ride lot - Whether it is at the end of a line - Whether or not it connects to an airport			- Numbers of jobs - Population - Percentage of people with Bachelor's degrees - Household income - Pct of housing units owner-occupied - Percentage of land-use		- U.S. Census - Data from MAG employment - Data from Valley Metro - Data from Assessor's office parcel files
09	Sung and Oh (72)	- Number of bus routes - Average headways - Number of short bus routes - Number of bus stops - Distance between railway stations	- Percentage of the driveway - Total road length - Average road width - Four-way intersection density	- Number of railway stations that exists - Dead-end road	- Residential density - Commercial density - Business density - Land-use mix index for four land uses - Commercial/business mix index	- Three downtown subway accessibility - Regional subway accessibility - Average building group area - Average building area of each building	- Data from transportation operators - Data from government - Data from Smart Card
10	Ivan et al. (73)	- Frequency of train services - Daily frequency of urban transport - Daily frequency of suburban buses - Car parking capacity			- Population - The number of workers in service/secondary - Core urban area - The number of flats - Land prices - Rate of unemployed with basic education		- Transportation timetable data - Census data - Data from government
11	Song and Deguchi (74)	- Number of railway lines - Number of bus routes - Number of bus stops - Number of passengers in the station - Number of community bus routes - Number of nearby railroad stations in the living area			- Number of residents - Proportion of households living in dwellings over six stories - Number of detached households - Total floor area of buildings - 10-year rate of change (passengers, population of hinterland, number of employees, total building floor area, population over 65 years old, working-age population) - 15-year change in the number of public facilities - Number of facilities (large and small retail, public) - Population ratio of 65 years old and over - Average number of employees per business		- Field investigation - Spatial datasets
12	Kamruzzaman et al. (43)	- Public transport accessibility level		- Intersection density - Cul-de-sac density	- Net employment density - Net residential density - Land use diversity		- Transport timetable data - Census data - Survey questionnaire data - Spatial data-sets (websites)
13	Singh et al. (75)	- Quality and suitability of streetscape for cycling		- Quality and suitability of streetscape for walking - Density of controlled intersections/street crossings	- Residential/employment/ commercial density - Land use diversity - Level of mixed-ness of land uses - W.R.T residential land use	- Private investment in the area - Number of business establishments - Tax earnings of the municipality - Unemployment levels	- Data from ESRI Nederland Statistics Netherlands (CBS) - Open Street Map (OSM) -Google Maps - Other Spatial data in GIS
14	Monajem et al. (76)	- Frequency of train services - Number of stations within 45 min of travel - Passenger frequency - Proximity to the CBD			The same as Bertolini (47)		- Data from Tehran Urban Planning and Research Center and Tehran Railway Operation
15	David (50)	The same as Bertolini (47), except that the research scope was different for some indicators		- The pedestrian shed ratio	The same as Bertolini (47)		- Lisbon transport operators data - Census data - Open Street Map - Google Earth
16	Guowei Lyu et al. (48)	- Number of directions served by metro - Number of directions served by bus - Daily frequency of metro services - Number of stations within 20 min of travel by metro - Travel times to major employment and activity centers by metro		- Average distance from the station to jobs - Average distance from the station to residents - Length of paved footpath per acre - Intersection density - Average block size - Walk Scores	The same as Bertolini (47) Add: - Number of jobs Lack: - The number of workers in Economic cluster 3: industry and distribution		- Data from Beijing Mass Transit Railway Operation Corporation LTD. - Open Street Map - POI data grabbed through Baidu Maps Place API - Google Route Planner - Google distance API - Spatial datasets
17	Loo and du Verle (56)	- Levels of other public transit services - Convenience of other public transit - Road connectivity - Road density - Expressways		- Covered walkway - Total number of station exits - Open space	- Population density - Employment density - Residential development intensity - Commercial development intensity - Mixed commercial-residential development intensity - Overall development intensity	- Comprehensive development - Diversity of land uses - Household size - Diversity of housing types - Income - Retail	- TCS data for 2011 - The latest Travel Characteristics Survey data
18	Rodriguez and Vergel Tovar (77)	- On-street parking - Distance to CBD - Vacant and BRT - Segment on BRT		- Green areas density - Park density - NMT friendliness	- Public facility index/density - BRT-oriented facility index - BRT-oriented facility density - Land use index - Percentage of different functions - BRT-oriented land uses - BRT unsupportive land uses	- Percentage of different density, consolidation, and condition - High-rise development - Commercial and parking - Entropy - Population density - Segment density	- Field visits - Data from government - Data from Traffic Management Unit
19	Singh et al. (34)	- Passenger load at peak hours - Passenger load at off-peak hours - Frequency of transit service - Interchange to different routes of the same transit - Interchange to other transit modes - Access to opportunities within walkable distance - Parking supply-demand for cars/four-wheelers or for cycles		- Total length of walkable/cyclable paths - Intersection density - Impedance pedestrian catchment area	- Population density - Commercial density - Land use diversity - Mixedness of residential land use with other land uses - Density of business establishments - Safety of commuters at the transit stop - Information display systems		- Data from the City Region - Data from the Statistics Nederlands - Data from transit website - Data from ESRI Top 10NL - Data from Open Street Map
20	Renne (78)	- Average vehicle ownership - Transit commuting mode share		- Walk and bike commuting mode share	- Jobs density - Percentage of professional jobs/ service jobs/ other jobs - Average vehicle ownership		- The National TOD Database - The Transportation Database - 2010 Census and 2009 LED data
21	Zhao et al. (79)	- Density of the branch ways - Metro service interval - Number of bus lines - Car ownership			- Residential households - Land use entropy - Area of non-residential land - Housing price - Percentage of Income <5,000 RMB/month - Housing ownership - Average number of weekly shopping/dining/recreation trips		- Survey data - Baidu map
22	Gu et al. (80)	- Line density - Station density - Expressway density - Number of parking facilities	- Distance to passenger transport terminal - Number of bus lines - Number of bus stops - Distance to municipal public service facilities	- Street network density	- Urban land coverage ratio - Urban population coverage ratio - Population density - Employment density	- Density gradient - Land use mix - Job-housing imbalance - Ground-floor retail density	- National Climate Center - China Statistical Yearbook - Beijingcitylab.com - Geo-spatial data (include POI) from Baidu Map geographical databases - National Census Data - The Worldpop database
23	Zhou et al. (39)	- The average number of stops from an M-SA to others - The overall accessibility of a station in the metro network - The ratio of the actual travel distance and the straight-line distance between one metro station and all other stations - The number of bus stops in the subarea		- The ratio of daytime population to a nighttime population in the subarea - The total numbers of daytime and nighttime population in the subarea	- The ratio of the number of POIs in the subarea - The ratio and percentage of the number of restaurants and gated-community, POIs in a given M-SA to that of all the other M-SAs in the subarea - The percentage of gated-community POIs in the subarea		- Weibo check-in's - Weibo POI data - OpenStreetMap
24	Li et al. (63)	- Metro station bearing capacity - Metro station service capability - Metro station connectivity - Accessibility(Average distance to different places)		- Walkability (Proportion of walkable blocks, Walking distance from the station, Average walkability of residential quarters)	- Land use diversity - Land use mixture		- Shanghai Metro official website - Baidu traffic big data - POI
25	Zhou et al. (55)	- Regional metro accessibility - The average number of Intermediate stations - Network directedness - Time and distance to CBD - Car walkability - Bikeability - The number of bus stops and metro stations - Whether the station belongs to two special lines	- Whether the station belongs to two commute lines from the center to the exurb - Whether the station opened before 2004, after 2012 - Whether there are one or more urban villages in a metro station area		- All destination intensity - Destination intensity (retail) - Destination intensity (entertainment) - Destination intensity (restaurant) - Destination intensity (residence)	- Simpson index - Whether the station is in Futian or Luohu (CBD) - Whether the station is in Longhua (the youngest administrative district in Shenzhen)	- Weibo POI - OpenStreetMap - Baidu heat Maps - Baidu Maps - The OfO company - Smart Card data
26	Zhou et al. (81)	- Mean custom distance - Network quantity penalized by distance - Two-phase destination - Betweenness	- Time to CBD - The number of bus stops - The number of shared bike users	- 30% Street-based accessibility - 50% Street-based accessibility	- POI - Whether the station is in CBD - Whether the station is in the Futian district - Construction phase	- Village (Whether there was at least an urban village) - Urban renewal (Whether there was at least an urban renewal project)	- Weibo POI - Open Street Map
27	Su et al. (65)	- Carrying capacity of metro station - Serving capacity of metro station - Topological connectivity of metro network		- Walkability (Intersection density, Walk score, Street connectivity)	- Land use diversity - Land use mix - Serviceability - Accessibility		- The local metro companies. - The local Institute of Surveying and Mapping.

*CBD; commercial business district; PoIs: Points of Interest; TOD: Transit-oriented development*.

Although many studies have begun to employ quantitative approaches to investigate the relationship between metro station accessibility and neighborhood vitality, a majority of them still rely on the traditional manually collected data. Thus, there is a need for a more systematic and human-oriented approach to evaluate TOD performance in metro station areas, especially in high-density cities. In this study, based on multi-source data and quantitative approach from the pedestrian-oriented perspective, we opted to combine the pedestrian-friendly dimension with T and D. With reference to earlier studies, we considered specific pedestrian-friendly features of the “oriented” dimension that can receive interventions by policymakers, urban planners, or designers as practical TOD planning tools.

## 3. Data and Methods

### 3.1 Analytical Framework

**Figure 1:**
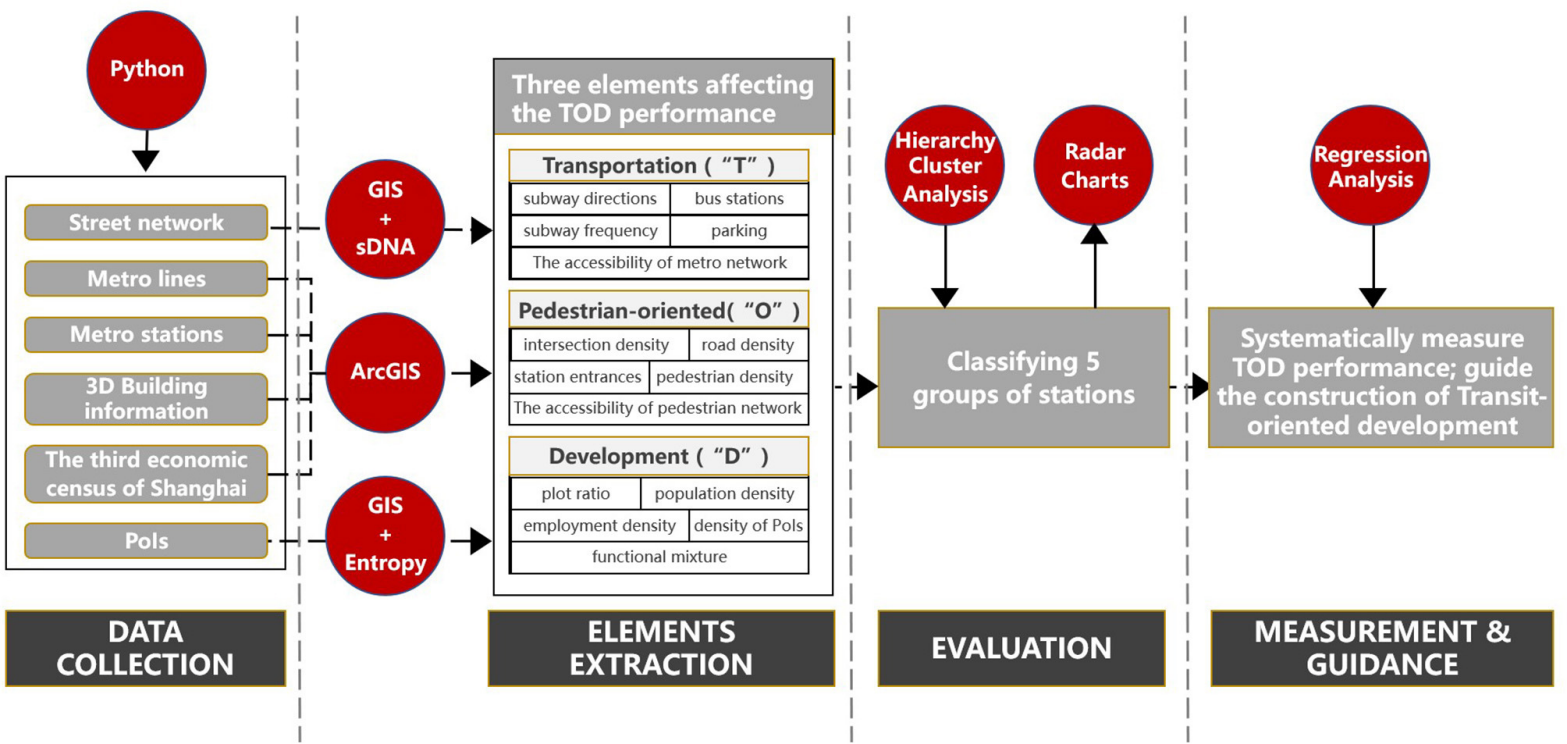
Analytic framework.

The four key steps of the present study were data collection, element extraction, evaluation, measurement, and guidance (Figure 1). First, metro lines and stations, 3D building information and PoIs, the third economic census, and the street network of Shanghai were gathered. Second, three critical TOD performance variables were extracted from the dataset. Each dimension was evaluated by five sub-indicators, the calculation details of which will be discussed later. Third, the evaluation process had two stages: (1) to create five TOD performance categories, hierarchical cluster analysis was utilized to organize the dataset into a cluster tree; (2) radar charts were then used to directly display numerous quantitative factors for each type. Therefore, a TOD performance measurement system was created to provide planners and policymakers with a better understanding of existing TOD and to diagnose common problems and design targeted policies for special station types.

### 3.2 Study Area: Shanghai

Shanghai, China's metropolis, has what is officially the longest railway network worldwide (59). According to the Shanghai Transportation Industry Operation Bulletin, as of June 2021, the city has completed 460 metro stations and 19 lines, with a total operational length of 772 km. The metro railway is Shanghai's major mode of transportation, carrying over 10 million passengers each weekday. However, the performance and types of stations vary significantly, as do the surrounding land use and morphology. Thus, Shanghai provides a sufficient number of cases, and it is essential to analyze the TOD variants and typology to discover common problems and offer targeted strategies.

Local governors have introduced the TOD concept to land-use planning and adopted the TOD as a critical strategy for sustainable urban development. Recently, the Shanghai municipal government announced the Fourteenth 5-Year Plan for Shanghai Comprehensive Transportation Development, which emphasizes station-city integration as a more specific requirement for metro station construction. In particular, building a systematic and comprehensive transportation system for new towns is highlighted in this plan to achieve internal high-quality traffic (60). In addition, Shanghai has outlined its plan for digital transformation, aiming to become a global capital for digitization by 2,035. Therefore, a data-driven approach to systematically measuring TOD performance seems urgent for understanding and promoting better place-making.

### 3.3 Measuring TOD Performance *via* Three Dimensions

**Figure 2:**
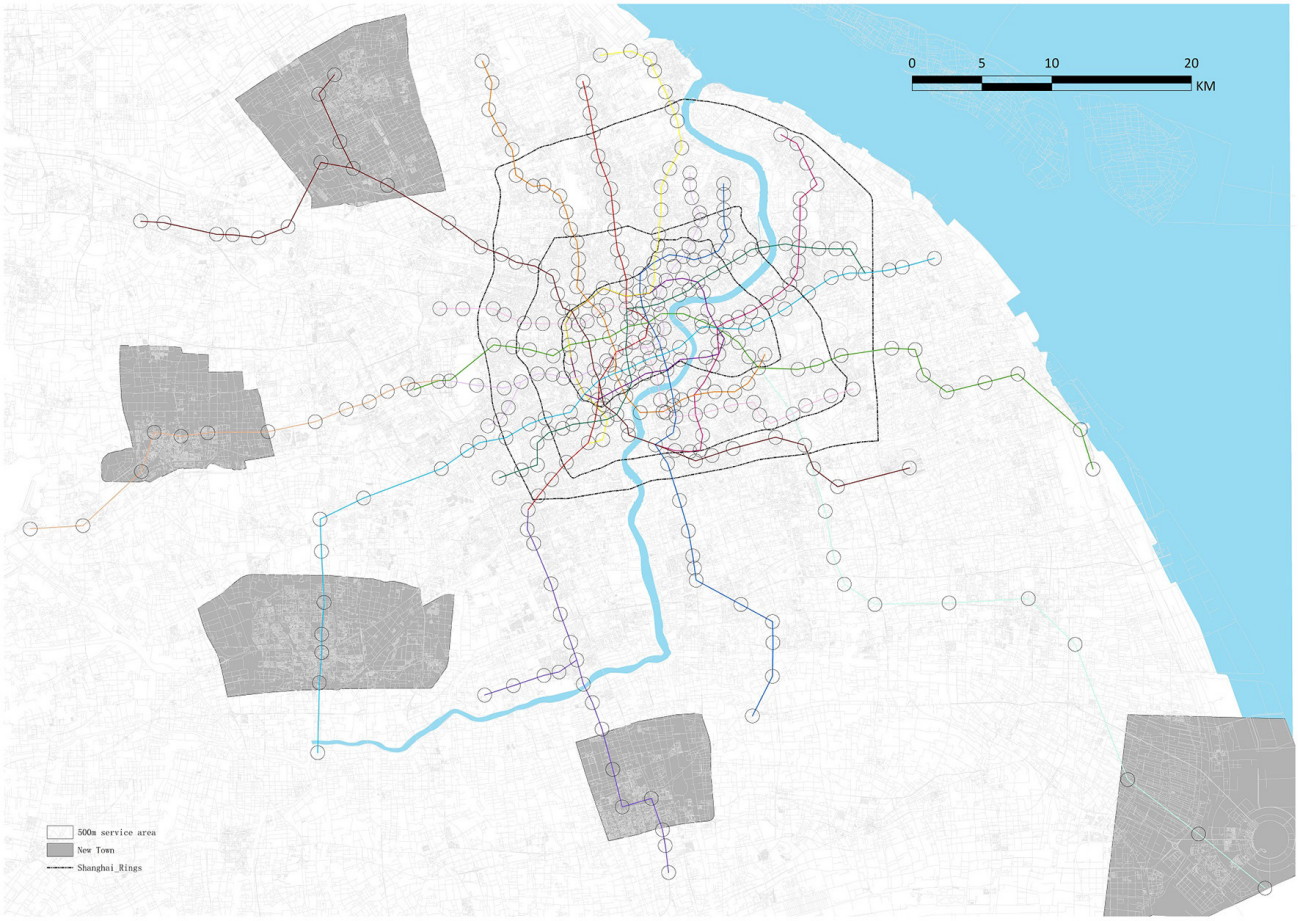
The study area and 500 m metro service area.

This study used Shanghai's existing metro stations in 2019 as cases, which comprised 347 metro stations and 18 metro lines (Figure 2). In accordance with the TOD theory and comfortable walking distance in Shanghai (9), the research scope (RS) of the built environment surrounding each metro station was within a 500-meter radius. In the context of a Chinese city, Lyu (48) conducted a systematic review of TOD indicators and divided them into three categories. Considering the occurrence frequency of each indicator in earlier literature (Table 1) and their data availability, we selected 15 indicators from three dimensions, “Transportation (T),” “Pedestrian-oriented (O),” and “Development (D),” to measure the TOD performance of the aforementioned stations (Table 2). The T dimension represents transport development in metro station areas, D dimension reveals the land use development, and O dimension represents the pedestrian-oriented functional and morphological characteristics in metro station areas.

**Table 2:** Overview of indicators.

**Indicator**	**Indicator description**	**Data sources**
T1 Number of metro lines	The number of rail transit lines serving the site	Shanghai metro official website
T2 Metro frequency	Train frequency during non-peak hours at the station on weekdays	Shanghai metro official website
T3 Accessibility of the metro station	The average betweenness centrality value of the lines connecting to the station	Shanghai metro official website
T4 Number of bus stations	Number of bus stops within 500 m of the station area	Gaode PoIs
T5 Number of parking lots	The number of parking lots within 500 m of the station area	Gaode PoIs
O1 Density of road network (to represent block size)	The density of the road centerline within 500 m of the station area	OSM
O2 Density of pedestrian network	Pedestrian road network density within 500 m of the station area	Baidu network
O3 Accessibility of pedestrian network	Average betweenness centrality value within 500 m of the station area	Baidu network
O4 Entrance and exit	Exits number of each station	Shanghai metro official website
O5 Intersection density	Intersection density of road centerline within 500 m of the station area	OSM
D1 FAR (floor area ratio)	The ratio of the gross floor area of buildings and the total buildable area	Baidu built environment data (building plots)
D2 Density of PoIs	The density of PoIs within 500 m of the station domain	Gaode PoIs
D3 Function mixture	Diversity of PoIs within 500 m of the station domain	Gaode PoIs
D4 Employment density	Employment density within 500 m of the station area	Third Economic Census in 2013
D5 Population density	Resident population density within 500 m of the station area	Population census of Shanghai in 2010

*PoIs, Points of Interest*.

For three dimensions, with reference to the earlier literature (25, 48, 52), we first chose the indicators “Number of metro lines,” “Metro frequency,” “Accessibility (betweenness) of the metro station,” “Number of bus stops,” and “Number of parking lots” for T dimension; “Density of road network,” “Intersection density,” and “Number of entrances and exits” for O dimension; and “Function mixture,” “Employment density,” “Population density” for D dimension. Second, to show an overall character of walking capability around the metro station, we introduced new indicators to provide a more precise perspective; both O2-Density of pedestrian network and O3- Accessibility of pedestrian network were calculated by sDNA (61), based on pedestrian network from Baidu Map. Third, with the help of built environment data, we used “Floor area ratio and Density of PoIs” to enrich the “D” dimension.

#### 3.3.1 Dataset

Multi-sourced urban data, including traditional and non-traditional data, were collected to represent aforementioned indicators. For example, on the one hand, for non-traditional data, 77,724 street polyline from Open Street Map (OSM) (2019), 559,777 street polyline and 754,607 building plots data from Baidu maps API and Python (2019), and a total of more than 2.7 million PoIs data from Gaode Map (2019) were collected. On the other hand, traditional data provided by Shanghai metro official website and Shanghai census was used to represent the physical characteristics of metro stations and social attributes of surrounding blocks.

Then, further data preprocessing was conducted in ArcGIS. For building plots data, each building with a height below 3m or over Shanghai's highest building (640 m) was removed. For PoIs data, we deleted some unimportant information, retaining only the name, geographical location, and multilevel categories' fields. Next, repetitive records and any record with publication time after July 16, 2019 were deleted. As for multilevel categories of this PoIs dataset, we used the second-level category to sift out bus stations and parking plots. The top-level category, used to calculate function mixture, was grouped into nine categories according to the types of residents' daily activities and the actual functions of each PoIs, including residential communities, traffic facilities, commercial and business, tourist attractions, food and shopping, education facilities, government and public services, financial services, and public facilities (62). Among them, those in the commercial and business categories were replaced with the same type of Baidu PoIs, since each Gaode PoI in this category represents one shopping mall rather than an individual retail store by Baidu PoI.

Thereafter, all datasets were intersected with RS separately. As a result, 65,981 polylines and 154,480 building plots of Baidu Map, 20,465 polylines of OSM, and 169,168 PoIs of Gaode Map were extracted. In the finalized data analysis, which is demonstrated below, the resultant data matrix had 347 rows, one for each station, and 15 columns, one for each indicator.

#### 3.3.2 Transportation Dimension

As previously stated, the T dimension was used to depict the station's convenience for automobiles and the surrounding environment. The number of metro directions, bus stations, and parking spaces, along with metro frequency and metro network accessibility, were chosen to represent the “T” component. For example, the number of metro directions and metro frequency can indicate metro station size, whereas the other three indicators can indicate how convenient it is to get to this station by vehicle.

**Figure 3:**
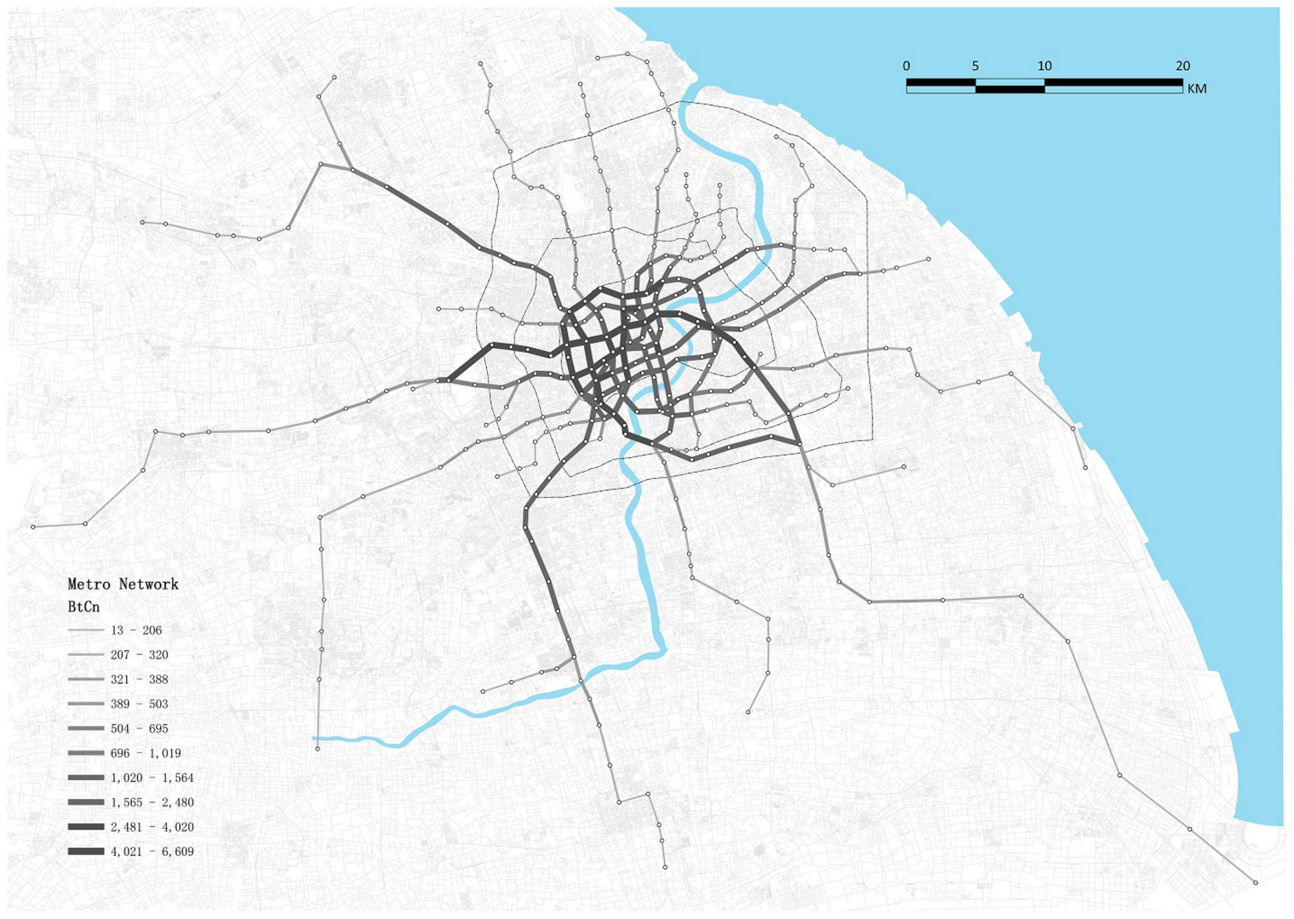
Betweenness centrality of Shanghai metro network.

The number of metro directions and metro frequency were obtained from Shmetro's official website (63), which is the administrative authority for Shanghai's metro railways. The number of metro directions distinguishes terminal stations; however, the number of metro lines obtained directly from the website does not. The metro frequency indicated the number of metro trains that would run through the station per hour. Python and AutoNavi's Map API were used to capture 2,764,864 PoIs. Following that, the numbers of bus stations and parking spaces, which are typically impossible to estimate manually, were indicted by the number of matching functions of PoIs within a 500-meter radius of each station. The measure of betweenness centrality of each line between two stations was calculated by sDNA (61) to reflect the flow potential of each link of the metro network (Figure 3). Betweenness centrality was measured using the following equation:


(1) $$betweenness\left(x\right)=\sum_{yz}n_{yz}^{x}$$

where $n_{yz}^{x}$ is 1 if x lies on the shortest path from y to z and 0 if it does not.

The betweenness centrality of each station is the average value of links within each station area.

#### 3.3.3 Pedestrian-Oriented Dimension

The O dimension was used to reflect the station's pedestrian accessibility and the surrounding environment. Similarly, the density of the road network, pedestrian network, and intersections, and the number of metro station entrances and accessibility of the pedestrian network were chosen to represent the “O” component. For example, the number of metro station entrances can demonstrate how many ways citizens can walk to the station, whereas the other four indications can illustrate how convenient it is to reach the station on foot.

**Figure 4:**
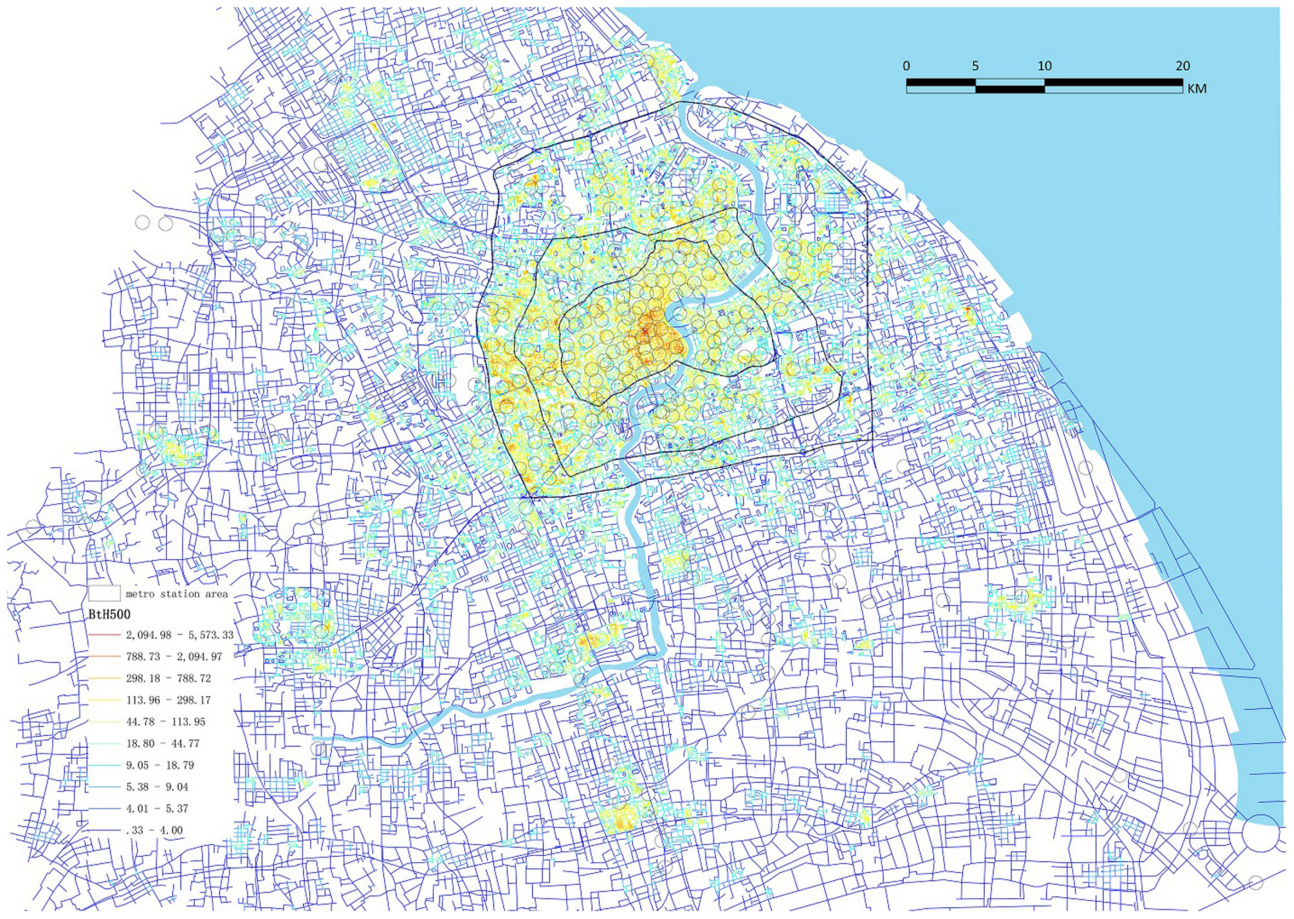
Hybrid betweenness centrality of the pedestrian network by the software sDNA (radius at 500 m).

Except for the number of metro station entrances, which was obtained from Shmetro's official website, the other four indicators were calculated on ArcGIS, based on the road network from the Open Street Map (OSM) platform and the pedestrian network from Baidu; the later dataset contains more branches than the earlier one. Specifically, road network density was calculated by dividing the total length of the main roads in an RS by its area; the density of the pedestrian network and intersections were the total length and the intersection number of pedestrian roads in an RS over its area. Finally, as shown in Figure 4, the accessibility of the pedestrian network was captured by the betweenness hybrid (BtH500) of pedestrian network use, sDNA (37).

#### 3.3.4 Development Dimension

The final component was the D dimension, which was used to describe the development level of the station's surrounding built environment. The plot ratio, density of PoIs, functional mixture, employment density, and population density, which is more connected to society and economics, were chosen to symbolize the “D” component. Plot ratio and employment/population density, for instance, can be used to demonstrate the density of people and buildings inside the RS. The other two indicators, the density of PoIs and functional mixture, can substantially define its diversity.

**Figure 5:**
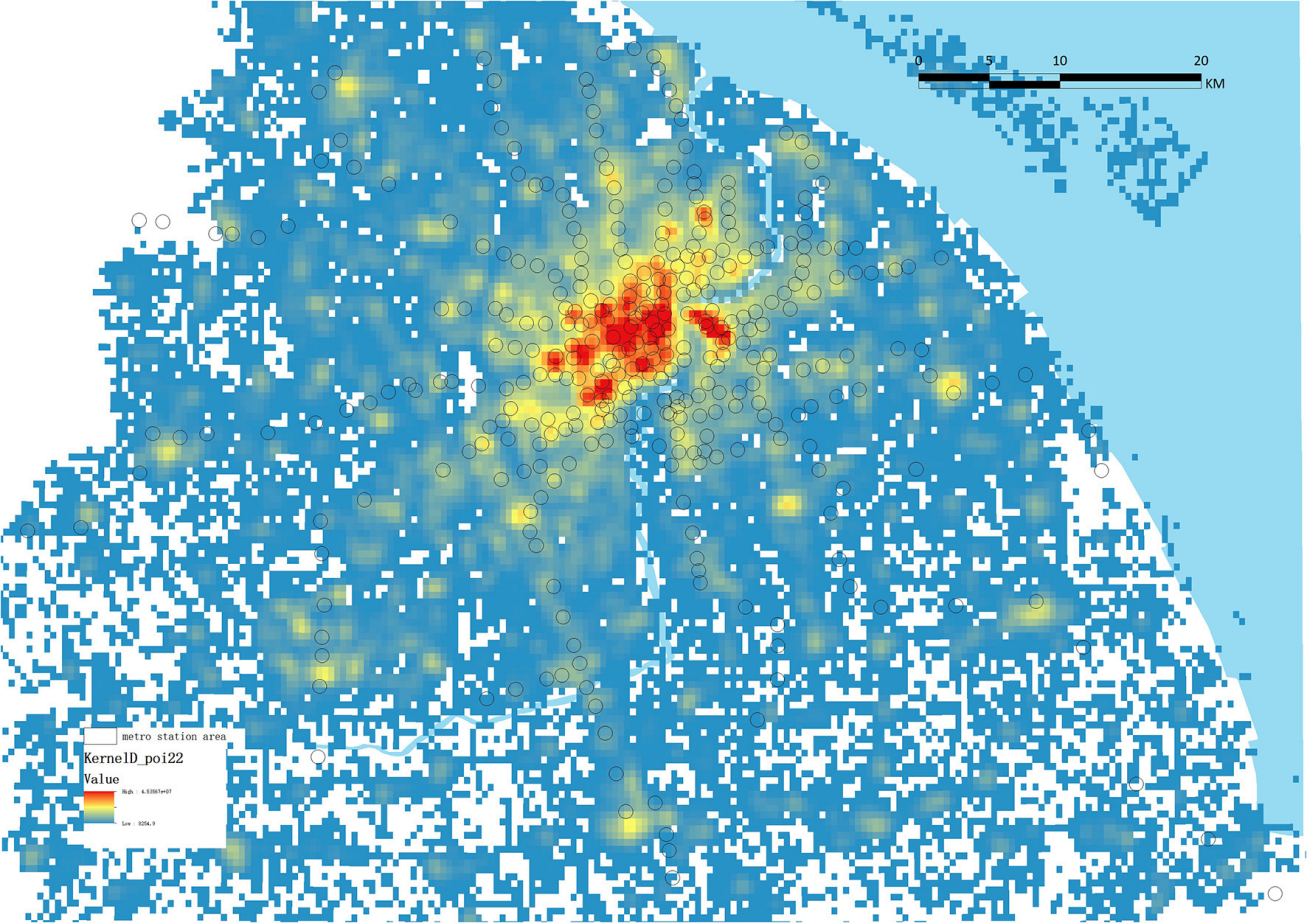
Example of nuclear density of PoIs.

These five indicators originate from various sources. First, the footprint and height information of buildings of 754,607 buildings plots were retrieved via Baidu maps API and Python to get FAR (floor area ratio). Second, the total number of PoIs over the range of the relevant RS was used to calculate PoI density (Figure 5). Further, the functional mixture was quantified using the Shannon entropy of the nine categories of PoIs (64). The formula used is as follows:


(2) $$SW_{i}\;=\;-\sum_{i=1}^{R}p_{i}\;*\;\ln\;p_{i}$$

where *SW*_*i*_ represents the Shannon–Wiener index of each station area, *P*_*i*_ is the proportion of urban facilities belonging to the *i*th type of functional categories, and *R* is the total number of main functional categories (9 in our analysis).

#### 3.3.5 Indicators Integration

To obtain the final integrated TOD performance, the abovementioned three components were added with equal weight. Before integrating all indicators, they were rescaled to have minimum and maximum values of 0 and 1, respectively, using the following formula:


(3) X_index =(X−Min(X))/(Max(X)−Min(X))

## 4. Results

### 4.1 Quantitatively Measuring TOD Performance

**Figure 6:**
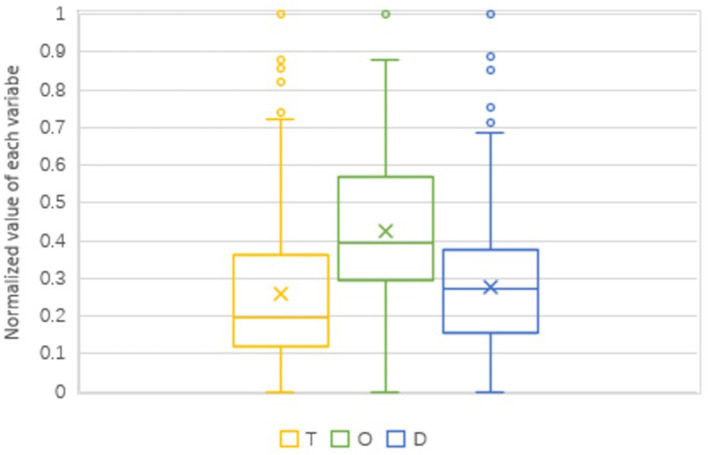
Boxplots for T, O, and D values (normalized).

In Figure 6, the box plots depict the descriptive statistics of various variables. The mean is represented by the *x* in the box, the median is represented by the line across the box, and the first (Q1) and third (Q3) quartiles are represented by the bottom and top of the box, respectively. The lengthy upper whiskers on all box plots imply that TOD performance varies depending on the variable's higher value. T and D's box plot positions are similar and low, showing that most of stations perform overall poorly on station-city integration.

**Figure 7:**
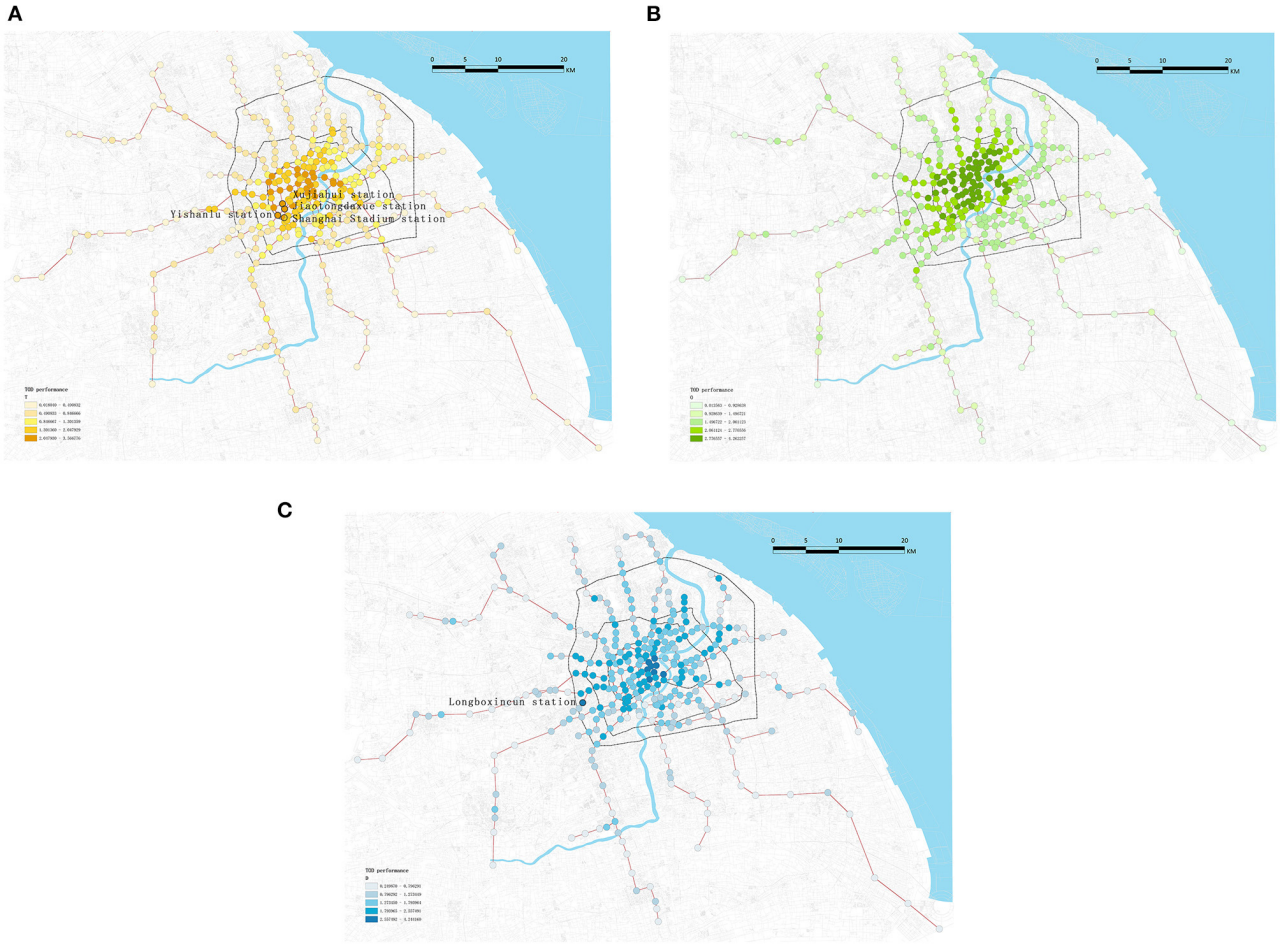
Visualization of **(A)** Transportation (T), **(B)** Pedestrian-oriented (O), and **(C)** Development (D) dimensions of TOD performance (dark color indicating high values and light color representing low values).

Each variable was rated and then divided into five classes by natural breaks (Jenks) to determine the geographical distribution of the imbalanced TOD performance. The lowest quintile received a 1, whereas the top quintile received a 5. Figure 7 displays the score of three components for assessing TOD performance at the metro station-level in Shanghai.

T component is shown in Figure 7A. The high values of the first and second grades are all within the inner ring, especially the stations of Lines 2 and 8 and those near the Lujiazui commercial business district (CBD) area. The north part of the Xuhui area is particularly notable, as its stations show high values despite its marginal location near the inner ring, such as Yishanlu, Xujiahui, Jiaotongdaxue, and Shanghai Stadium stations. This is mostly due to the fact that they are transfer stations with appropriate transportation facilities nearby.

As seen in Figure 7B, the number of the highest listed station for the O component is significantly larger than the other two. They were mostly evenly dispersed over the inner ring. Nonetheless, the most intriguing discovery was that the O values of the aforementioned unusual station in T and D were significantly reduced. Therefore, three variables of most stations are not in a coordinated development, while sharing the same characteristic of a decreasing tendency from the center to the periphery.

In comparison to T, D has fewer top-level values, but they are gathered more downtown (Figure 7C). Except for Longboxincun station, which is on the outer ring's boundary, almost all the top-ranked stations are in the most prosperous historical center. However, all these stations are surrounded by dense residential areas and CBDs. Another difference is that the second-highest stations on this map are significantly more dispersed than stations in the second-highest class of T component. This suggests that the degree of development inside the outer ring is reasonably even. This can also be seen in Figure 6, where the median and mean of D are almost equivalent, while the median and mean of T diverge the most.

### 4.2 Hierarchical Cluster Analysis

**Figure 8:**
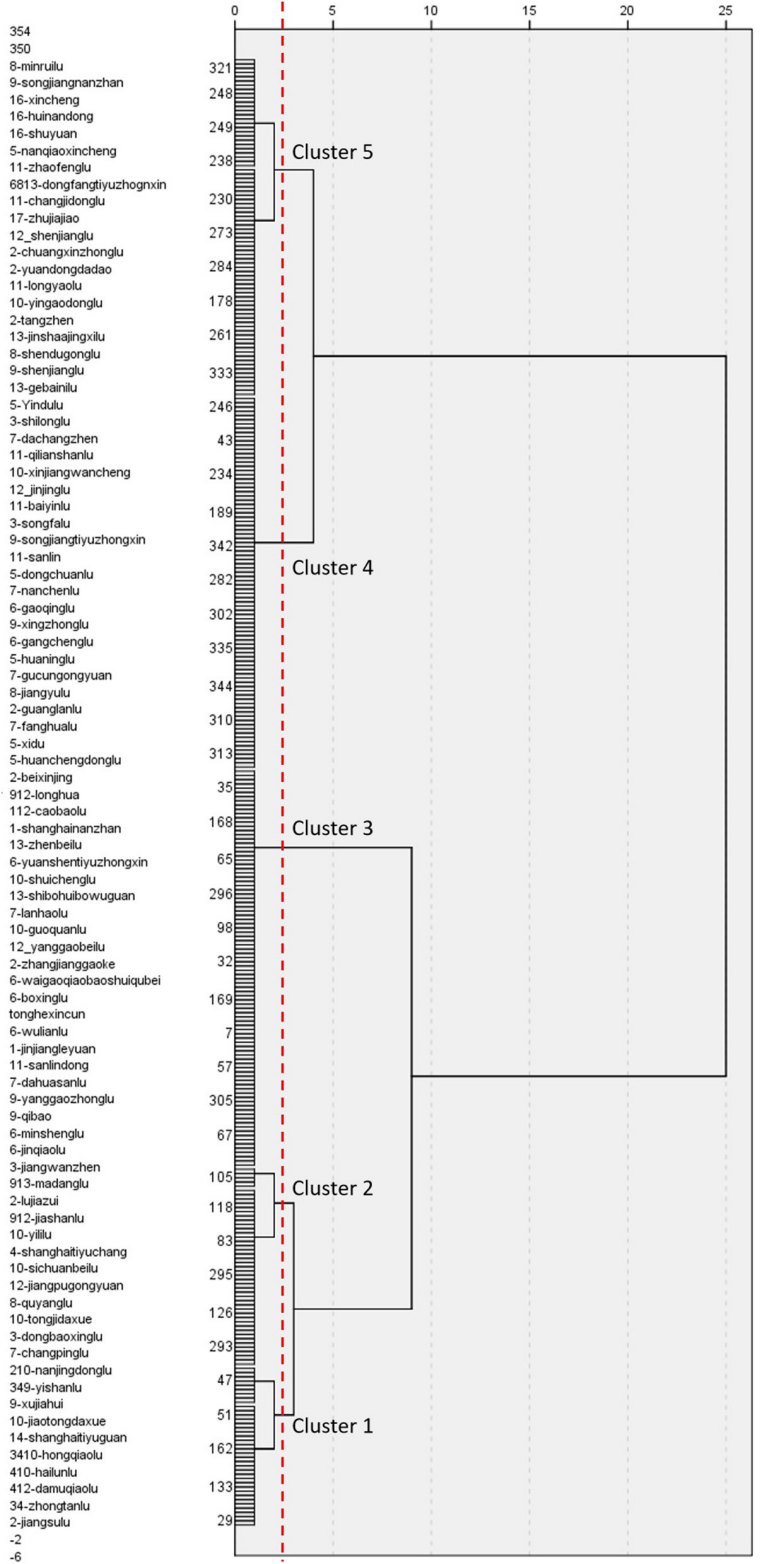
The hierarchical cluster analysis is based on three variables.

**Figure 9:**
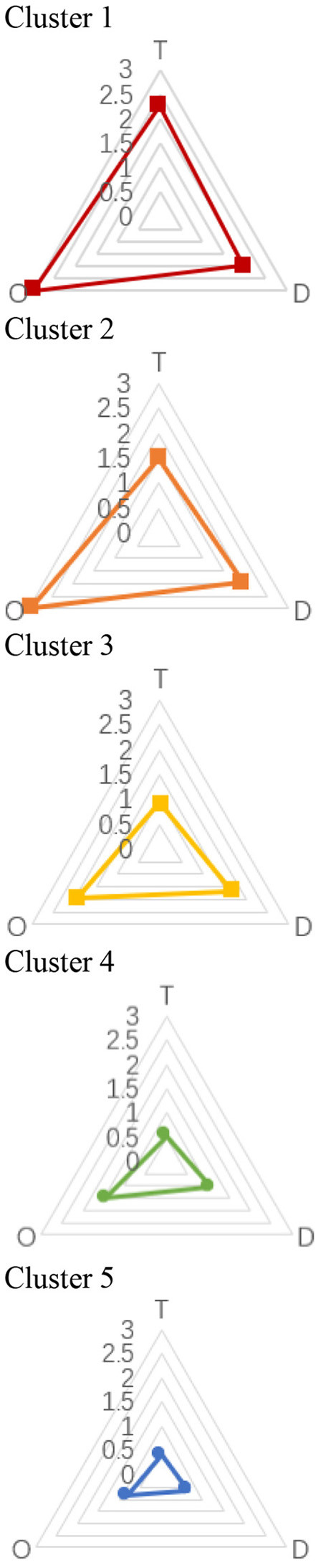
Rader charts for each variable by clusters.

**Figure 10:**
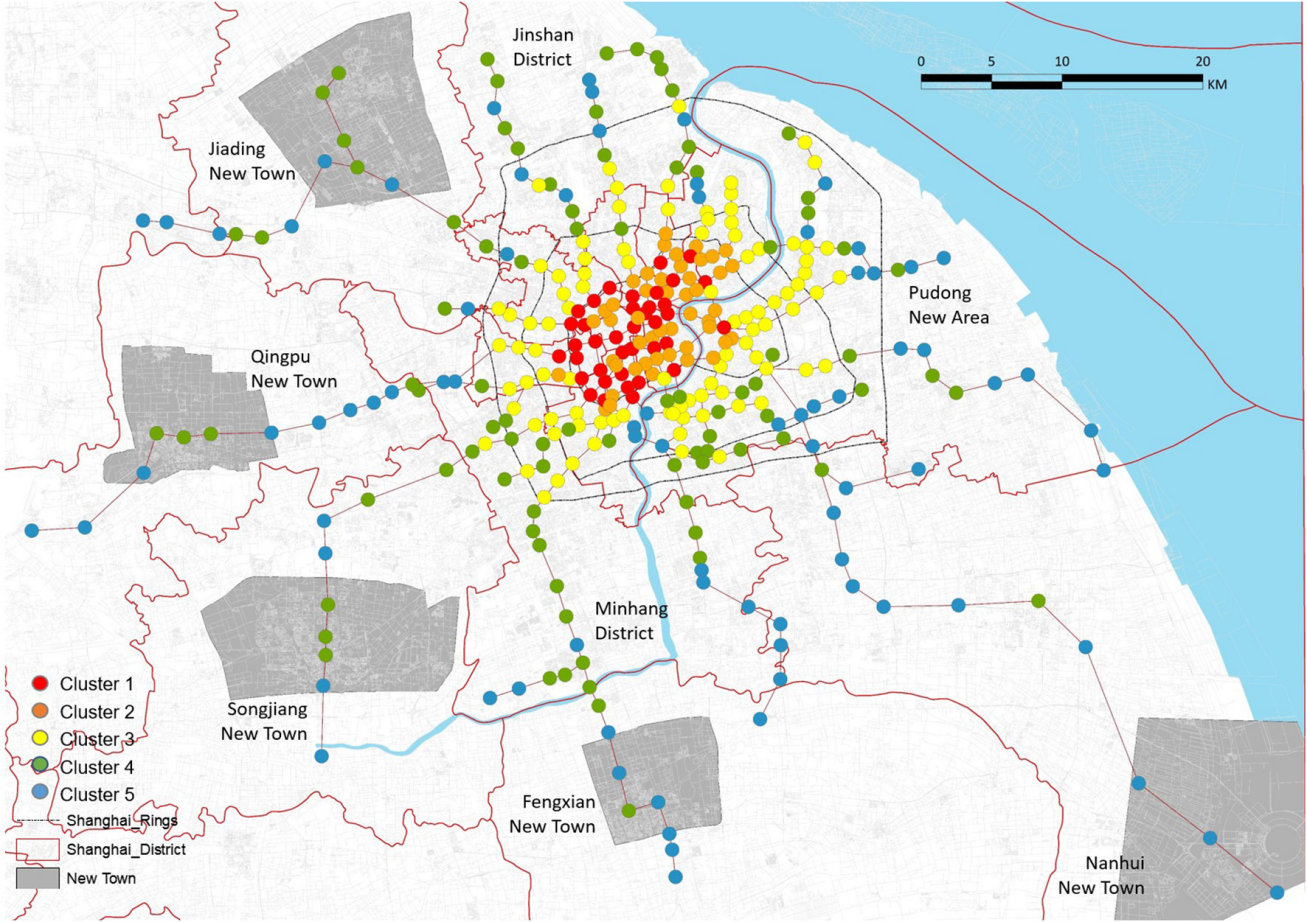
TOD performance by clusters in Shanghai.

A hierarchical cluster analysis, which is a multivariate statistical method for grouping cases according to the similarity of their characteristics, was performed to classify the data. In this study, we classified stations using all 15 variables. The dendrogram shown in Figure 8 summarizes the clustering process and reveals five representative clusters. This cutoff number was chosen to obtain more representative clusters. Then, we focused on the five clusters to identify similarities and differences between them. The radar charts in Figure 9 highlight the average ratings of major sections in TOD performance, allowing for visual comparison. Figure 10 depicts the number of clusters and their spatial distribution. When Figures 9, 10 are taken together, we can observe that each cluster had the following key characteristics:

Cluster 1—high TOD performance: The results for the three indicators for stations in these clusters were excellent (>2.0). T, O, and D all scored well in these groups, suggesting that the three variables were fairly balanced. Except for Shijidadao station in Pudong district, they are all situated at the center of the old town. Other stations in Pudong, although closer to the Lujiazui Financial Centre and Huangpu River, showed poor performance. This means that the busy areas may be uneven in TOD performance, although they are economically developed.

Cluster 2—relatively high performance except for T: The O and D indicators both showed reasonably high scores of 2.0 and 3.0, respectively. Variables in Cluster 2 are similar to those in Clusters 3 and 4 in terms of shape. In contrast to the other counterparts, the T component (1.5) was noticeably inadequate. This type of station could be found in the inner ring and surrounding Daxuelu area, Lujiazui area, Changshou, and Xujiahui road, which are Shanghai's subcenters. In this type, the transportation system could not sustain the exceeded growth intensity and vitality.

Cluster 3—medium performance: The three indicators in these clusters had mediocre scores (1.0 < scores < 2.0). The majority of them were found between the inner and outer rings. They also occurred in a consistent linear pattern. The cause for these unpleasant outcomes was similar to that in Cluster 2, but the built environment in their surrounding regions was poorer quality.

Cluster 4—relatively low performance, especially T: The three indicators for stations in this cluster had relatively low scores. Some of the stations of this type were in the middle ring, some were in the outer ring, and others were beyond the outer ring. However, the stations of this type beyond the outer ring of appeared where road network density suddenly increased. In addition, when we combined their spatial distribution with Shanghai's regional policy, some unexpected findings were revealed. The locations of these metro stations perfectly matched the core area of new towns in the Fourteenth 5-Year Plan (2021–2025). The plan contributed significantly to the growth of suburbs since the regions around these stations are equivalent to a quiet neighborhood in the inner ring. However, defects were still in evidence, which we will discuss in the next section.

Cluster 5—poor TOD performance: This cluster was fundamentally low-performing, with all indicators' ratings below 1.0. The vast of these metro stations are located in remote areas. In general, the south of Shanghai performed worse in TOD than the north, resulting in the city's uneven growth on a wider scale.

It is worth mentioning that we compared our results with previous research (51, 52), and found some differences. Our study, to some extent, is consistent with that of Li et al. suggesting that some metro stations in central areas are balanced (Cluster 1) in T and D. However, in reality, a number of central stations are not balanced (65). More than a half of the stations in the inner ring are Clusters 2 and 3. It is easy to notice in the radar chart that the T dimension is slightly lower than D in Cluster 2 and the overall TOD performance is relatively poor in Cluster 3.

## 5. Discussion and Conclusion

### 5.1 Shanghai Comprehensive Plan (2017–2035) and Implications for TOD Planning

The traditional core region of Shanghai, Puxi's seven districts, showed pretty good TOD performances. The TOD performance in Pudong New Area was the most unequal, with TOD categories diminishing from the inner to outer ring. Minhang and Baoshan were found to have the most Cluster 4 stations among the eight suburban districts, indicating that they are significantly more developed than the other six fringe areas. The northernmost district, Chongming Island, was excluded, since it did not have a metro station. As for the other areas, Shanghai announced a local Comprehensive Plan (2017–2035), which emphasizes the development of “Five New Towns,” including Qingpu, Fengxian, Jiading, Nanhui, and Songjiang. These new towns are envisioned as a comprehensive node city that prioritizes public services and living environment quality, representing a development shift from the urban core to new towns. In addition, the TOD categories mentioned above can also demonstrate the plan's effectiveness to some extent.

Differentiation can be noted between their TOD performance scores, although all new towns have relatively low performance. They only contain Clusters 4 and 5 stations. Moreover, the majority of Cluster 4 is located in the center of the new town. Those places were once satellite cities built 20 years ago. The numbers of type 4 station are, however, different in each of the five new towns. Notably, Jiading, Songjiang, and Qingpu each have more than three of this type, whereas Fengxian has only one, and Nanhui has none. This illustrates that, in comparison to other areas, Nanhui and Fengxian are inadequate in terms of population density, transportation, functional diversity, and living convenience. This is in line with the realities of spatial planning and transportation.

Therefore, targeted optimization guidelines should be introduced to different categories for their distinctive characteristics. For Clusters 4 and 5, which are the most potential categories, where their TOD surrounding areas are barely constructed, it is critical for the new territory development plan to integrate transportation planning and urban planning into the whole framework and make rather high-intensity constructions. For Clusters 2 and 3, where the T indicators are relatively low, planners can build new metro lines connecting them between the inner and the outer rings, reducing the inconvenience of a detour transfer. As for stations in Cluster 1, introducing more public areas and amenities could be beneficial for further improvement.

### 5.2 Transit Ridership, Transit Ridership per Capita, and TOD Performance

The correlation between ridership and TOD performance was analyzed to reveal the relationship between TOD performance and expected outcomes (Table 3). The correlation was analyzed with Pearson's correlation. Statistical significance was defined as a two-sided *p*-value of <0.05. Daily ridership data for 1 weekday (July 16) and 1 weekend day (July 20) of 2019 was used. As anticipated, the ridership on both weekdays and weekends increased with the TOD performance index. More specifically, the T value, which relates to transport infrastructure, was strongly correlated with daily ridership (*R* = 0.660 for weekdays and *R* = 0.634 for weekends). the O indicator was more strongly correlated with the ridership on weekdays (*R* = 0.612) than on weekends (*R* = 0.558). The D value, which measures the development of the station area, had a relatively lower correlation, with *R* = 0.580 for weekday and *R* = 0.551 for weekend, respectively. Moreover, comparing with daily ridership, the exiting ridership in morning peak hours has even stronger correlation with both T and O indicators, and has a slightly lower correlation (*R* = 0.543) with D value, indicating that the T and O dimensions are more important for commuters in choosing to ride the metro than it is for other types of train riders. This was in general consistent with existing studies, such as Zhou et al. (39).

**Table 3:** R correlation between ridership, ridership per capita, and TOD performance.

		**T value**	**O value**	**D value**	**TOD** **performance**	**LN** **resident** **density**	**LN** **employment** **density**
LN Daily ridership	weekday	0.660^**^	0.612^**^	0.580^**^	0.669^**^	0.431^**^	0.514^**^
	weekend	0.634^**^	0.558^**^	0.551^**^	0.629^**^	0.367^**^	0.429^**^
LN ridership in morning peak hours (7–9 am)	Morning peak	0.525^**^	0.501^**^	0.474^**^	0.543^**^	0.361^**^	0.406^**^
	Exit_peak	0.706^**^	0.660^**^	0.543^**^	0.694^**^	0.470^**^	0.677^**^
	Enter_peak	0.053	0.098	0.159^**^	0.110^*^	0.121^*^	−0.076
LN Daily ridership per capita	weekday	−0.096	−0.340^**^	−0.201^**^	−0.246^**^	−0.658^**^	−0.347^**^
	weekend	−0.087	−0.340^**^	−0.191^**^	−0.234^**^	−0.654^**^	−0.377^**^

* *Significant at 0.1 level*;

** *significant at 0.05 level; TOD, Transit-oriented development*.

We then explored the impact of TOD performance on ridership per capita. The indicator of ridership per capita had a high, strong correlation with resident density, with *R* = 0.658 for weekdays and *R* = 0.654 for weekends, indicating that the population plays a pivotal role in metro passenger traffic. In addition, employment density, O value, and D value were significantly correlated with ridership per capita. Among them, people's willingness to travel by metro was more affected by job density and O value. No significant correlation was found between T value and ridership per capita, indicating that the impact of T value on ridership relies on population density.

### 5.3 Contributions and Limitations

In high-density cities, such as Shanghai, TOD construction is a critical component for both economic growth and daily wellbeing. The classic node-place method is unable to achieve the pedestrian-friendly and human-oriented goals of a sustainable city. By incorporating the pedestrian-oriented dimension in the classic “node-space” method and 5Ds framework, this study provided a comprehensive framework for measuring TOD performance.

Although various supplementary methods have been conducted to measure the accessibility of pedestrian networks, few of them have shown the overall character of walking capability around the metro station. Unlike previous studies, this study offers a data-driven and more efficient approach toward conducting an inclusive and fine-scale framework to measure TOD performance. With a combination of traditional data collected by an official government department, high-resolution open data, and innovative technology, large-scale analyses will be easy to conduct. The method can be quantitatively achieved from the pedestrians' point by combining new techniques with more precise datasets, such as the pedestrian network, the metro ridership, and 3D built environment data. Moreover, the findings of this research were summarized and described in radar charts, box plots, and colored maps, making the structured analysis easy to understand, providing an operable and effective methodology to support urban planners and policymakers by visualizing the results with the ongoing Shanghai Comprehensive Plan (2017–2035). The simple visualized results can also be shown to communities with a developing application on mobile phone, allowing the TOD performance to be assessed using public awareness.

In the future, we can further or enhance this study in the following aspects: First, the indicator of the O dimension is insufficient. New urban data and technology can be examined, such as SVIs and emotion sensors combined with virtual reality, could reflect pedestrians' feelings more in-depth. Second, the numbers of workers and inhabitants were only represented by the official census data. Changes in population density are vital for future studies. Consistent location-based service data and mobile phone data can help with this information. Finally, TOD indicators are interdependent with each other when classifying its typologies (65). For follow-up research, experts in urban fields should be invited to verify the validity of the chosen indicators and determine their proportions by using the analytic hierarchy process.

## Data Availability Statement

The datasets analyzed for this study are included in the article/table, further inquiries can be directed to the corresponding authors.

## Author Contributions

LZ: conceptualization and funding acquisition. DQ and LZ: data collection, methodology, software, and writing—review & editing. DQ and XH: formal analysis and visualization and writing—original draft. All authors contributed to the article and approved the submitted version.

## Funding

This research was funded by the National Natural Science Foundation of China (52008297); Shanghai Pujiang Program (21PJC114).

## Conflict of Interest

The authors declare that the research was conducted in the absence of any commercial or financial relationships that could be construed as a potential conflict of interest.

## Publisher's Note

All claims expressed in this article are solely those of the authors and do not necessarily represent those of their affiliated organizations, or those of the publisher, the editors and the reviewers. Any product that may be evaluated in this article, or claim that may be made by its manufacturer, is not guaranteed or endorsed by the publisher.
